# A power quality disturbances classification method based on multi-modal parallel feature extraction

**DOI:** 10.1038/s41598-023-44399-7

**Published:** 2023-10-17

**Authors:** Zhanbei Tong, Jianwei Zhong, Jiajun Li, Jianjun Wu, Zhenwei Li

**Affiliations:** 1 College of Intelligent Systems Science and Engineering, Hubei Minzu University, Enshi, 445000 China; 2 State Grid Hubei Electric Power Company, Enshi Power Supply Company, Enshi, 445000 China

**Keywords:** Renewable energy, Electrical and electronic engineering

## Abstract

Power quality disturbance (PQD) is an important problem affecting the safe and stable operation of power system. Traditional single modal methods not only have a large number of parameters, but also usually focus on only one type of feature, resulting in incomplete information about the extracted features, and it is difficult to identify complex and diverse PQD types in modern power systems. In this regard, this paper proposes a multi-modal parallel feature extraction and classification model. The model pays attention to both temporal and spatial features of PQD, which effectively improves classification accuracy. And a lightweight approach is adopted to reduce the number of parameters of the model. The model uses Long Short Term Memory Neural Network (LSTM) to extract the temporal features of one-dimensional temporal modes of PQD. At the same time, a lightweight residual network (LResNet) is designed to extract the spatial features of the two-dimensional image modality of PQD. Then, the two types of features are fused into multi-modal spatio-temporal features (MSTF). Finally, MSTF is input to a Support Vector Machine (SVM) for classification. Simulation results of 20 PQD signals show that the classification accuracy of the multi-modal model proposed in this paper reaches 99.94%, and the parameter quantity is only 0.08 MB. Compared with ResNet18, the accuracy of the proposed method has been improved by 2.55% and the number of parameters has been reduced by 99.25%.

## Introduction

The environmental pollution caused by traditional fossil energy sources has forced mankind to develop cleaner energy sources. To promote sustainable development strategies, modern power systems use a lot of renewable energy represented by wind and solar energy^1^. However, the random and intermittent nature of renewable energy sources has triggered a series of PQD problems, resulting in transformer overheating, decreased product quality, reduced equipment life, and false tripping of circuit breakers, which affect the safety and stability of the power system^2^. Therefore, the problem of power quality needs to be addressed urgently. An important step in improving PQD problems is to accurately identify the type of PQD^3^.

The recognition of PQD consists of two parts: feature extraction and classification. Common feature extraction methods include wavelet transform^4,5^, empirical mode decomposition^6,7^, variational mode decomposition^8^, S transform^9,10^, etc. According to the characteristics of the appropriate classification algorithm, such as: support vector machine^11–14^, decision tree^15^, artificial neural networ^16,17^, etc. Literature^18^ proposed using discrete wavelet to extract PQD characteristics , then combining artificial bee colony and particle swarm optimization to select optimal features, and finally using probabilistic neural network to realize PQD classification. Literature^19^, the cuckoo algorithm is improved to optimize the penalty factor, relaxation variable and feature number of multi-class SVM, which improves the classification accuracy to a certain extent. Literature^20^, for the PQD of distributed new energy generators, the pyramid algorithm without wavelet transform is used to extract features, and then the random gradient lifting tree is used for classification, which effectively improves the classification accuracy. These methods have their own characteristics and have made significant contributions to the research of PQD classification. However, the artificially extracted features of the signals make the classification results susceptible to human interference.

With the advancement of artificial intelligence technology, some new methods have provided assistance in solving the classification problem of PQD^21,22^. The deep learning methods represented by convolutional neural networks (CNN) transform time-series signals into two-dimensional images, and then automatically extract spatial features from the images^23^. Literature^24^ used continuous wavelets to convert PQD into color images, and then used Bayesian CNN for classification. This method achieves certain results, but ignores the influence of some temporal features. Methods represented by recurrent neural networks (RNNs) extract temporal features from one-dimensional PQD signals and then perform classification^25^. Literature^26^ proposes introducing a dual attention mechanism in Bi-LSTM to increase the weight of important features, reducing computational complexity and improving accuracy. This approach effectively extracts the temporal features of the signal, but does not focus on the impact of spatial features on classification. Literature^27^ proposes a hybrid neural network model to convert PQD into a perturbed image, using CNN to automatically extract spatial features of the image, and then inputting the temporal features into a gated recurrent unit (GRU) for classification. This method extracts the spatial-temporal characteristics of the PQD signal, but may result in the loss of some temporal features during image conversion. Although the above methods can eliminate the interference of human factors, these single-modality models can easily lead to varying degrees of feature loss during feature extraction, which can have an impact on PQD classification. Meanwhile, in order to achieve better classification results, these deep learning models continuously increase the depth of the model by stacking, which makes the number of parameters of the model increase significantly and improves computational difficulty.

In recent years, inspired by the multisensory (visual and auditory) perception of the world by humans, research on classification methods has gradually shifted from unimodal to multi-modal domains^28^. Multimodal data fusion aims to combine different distributed and different types of data in a single space, including images, audio, and measurement signals^29^. Currently, it is mostly used in medical diagnostics, acoustics, and vision^30–32^, research in PQD recognition suffers from the problems of feature extraction methods and a large number of model parameters^33^. Multimodal information is obtained by fusing different modalities of information, and its amount of information exceeds that of a single modality. In previous studies, most of them adopted a single mode approach, that is, extracting features from one-dimensional signals or two-dimensional images. However, the types of PQDs in modern power systems are complex and diverse, and most single modal methods are prone to feature loss when extracting features, resulting in an inability to fully grasp the characteristics of the signal^34^. To address the above issues, this paper combines these types of data and proposes a PQD classification method based on multimodality LResNet-LSTM parallel feature extraction. The model simultaneously inputs the one-dimensional time-series signal and two-dimensional disturbance images of the PQD. LSTM and LResNet are used to extract the temporal and spatial features, which are then fused into a multimodal spatiotemporal feature (MSTF), and finally input into SVM for classification.

Based on the above research, this paper proposes a PQD classification method based on multi-modal LResNet-LSTM parallel feature extraction. The model uses LResNet and LSTM to extract spatial features and temporal features in parallel, and then fuses them into MSTF, which are finally input into SVM for classification.

The main contributions of this paper are as follows:

- This paper proposes a PQD classification model with multi-modal parallel feature extraction. The model designs spatial feature extraction (SFE) module and temporal feature extraction (TFE) module based on LResNet and LSTM respectively. The multi-modal feature fusion (MFF) module fuses the features extracted by SFE and TFE in parallel into MSTF to obtain more comprehensive feature information.
- A Light ResNet is designed based on the idea of residuals and depth-separable convolution. Compared to the traditional ResNet18, its structure is simpler and the number of parameters is greatly reduced. The use of Swish activation functions optimizes the classification performance of the model. After being fused with the temporal features extracted by LSTM, the accuracy of the model reached 99.94
- This article uses Cache to solve the problem of speed mismatch between SFE and TFE modules when running in parallel.
- Unlike traditional deep learning models that improve classification accuracy by increasing depth, the proposed model in this paper not only improves classification accuracy, but also achieves the light weight of the model.

The work in this paper is organized as follows: “Multi-modal PQD classification model” section proposes a multi-modal PQD classification model. “Simulation experiments” section verifies the effectiveness of the method through simulation experiments. “Conclusion” section is the conclusion.

## Multi-modal PQD classification model

### PQD classification model framework

**Figure 1:**
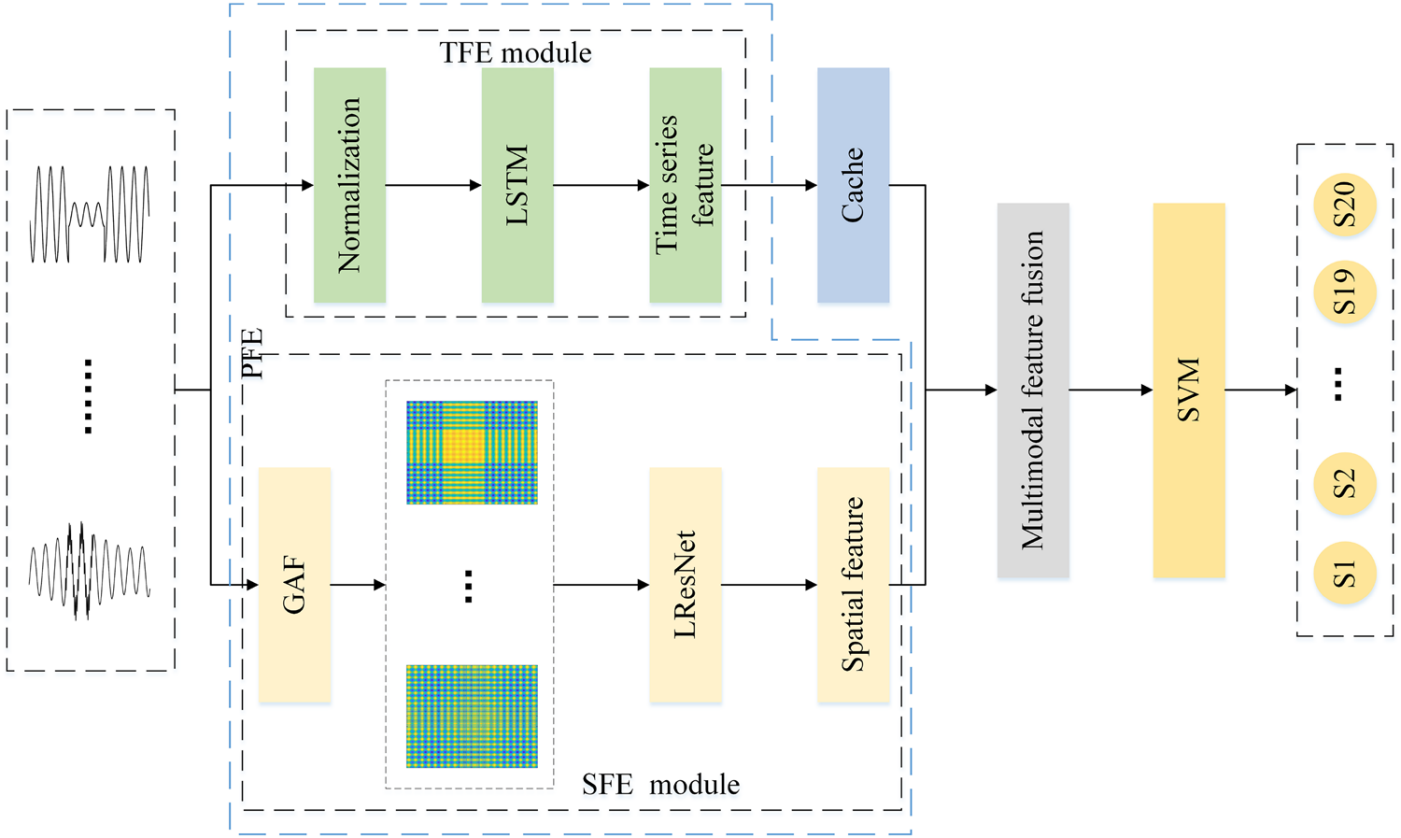
Multi-modal PQD classification model.

In this paper, we propose a multi-modal PQD classification model based on LResNet-LSTM, which mainly consists of a parallel feature extraction (PFE) module, an MFF module and a classification module. The model framework is shown in Fig. 1.

- Parallel Feature Extraction: PFE consists of two submodules: SFE and TFE. The SFE first transforms PQD signals into 2D disturbance images and then extracts spatial features using LResNet. TFE normalizes the PQD signal and extracts temporal features using LSTM. After this, TFE stores the features in the Cache and waits for SFE to complete its operation.
- Multi-modal feature fusion: This module splices the extracted spatial features and temporal features to obtain MSTF and outputs them to the next module.
- Classification: This module uses a better performing SVM as a classifier, first inputting MSTF for training, and then inputting the test set into the trained SVM for classification.

### Parallel feature extraction module

Due to the fact that LSTM needs to calculate the previous result before calculating the next result when processing sequences, it is unable to directly combine LSTM with LResNet into a parallel computing model. Therefore, we need to split the PFE module into two separate submodules: SFE and TFE. The two sub-modules can be used to extract the spatial features and temporal characteristics of the PQD signals respectively.

#### SFE module

SFE module consists of two parts: image conversion and feature extraction. Image conversion is done by gramian angular field (GAF), which maps the PQD signal into the polar coordinate system using the GAF method and converts it into a scrambled image by coding^35^. This method has been widely used to convert 1D signals into 2D images. The conversion process is as follows.

Step 1: The timing signal x(t) is mapped into the polar coordinate system. Where, is the angle and r is the radius.
  1 $$\begin{aligned} {\left\{ \begin{array}{ll}\varphi =\textrm{arccos}(x) \\ r=\frac{t_i}{N},t_i\in N \end{array}\right. } \end{aligned}$$
Step 2: Convert to Gram matrix.
  2 $$\begin{aligned} G=\left[ \cos \left( \varphi _{i}+\varphi _{j}\right) \right] =\left[ \begin{array}{ccc} \cos \left( \varphi _{1}+\varphi _{2}\right) &{} \cdots &{} \cos \left( \varphi _{1}+\varphi _{n}\right) \\ \vdots &{} \ddots &{} \vdots \\ \cos \left( \varphi _{n}+\varphi _{1}\right) &{} \cdots &{} \cos \left( \varphi _{n}+\varphi _{n}\right) \end{array}\right] \end{aligned}$$
Step 3: The imagesc function in MATLAB is called to implement the image conversion. Each element in the matrix specifies a pixel color in the image.

**Figure 2:**
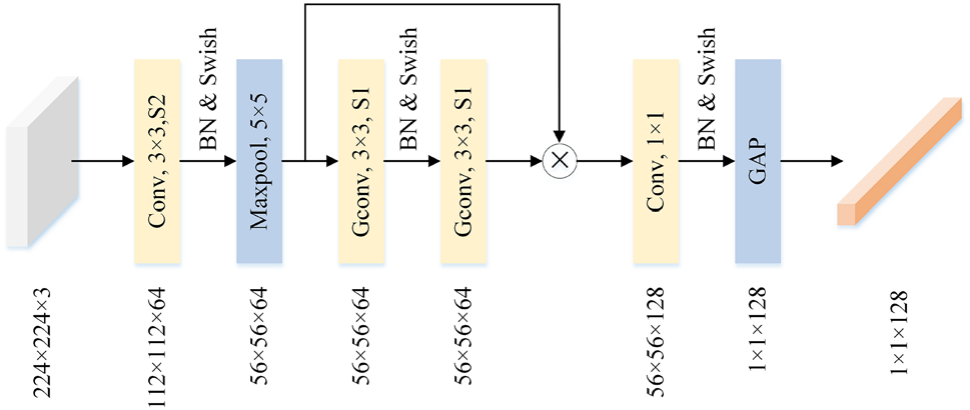
Structure diagram of LResNet.

This article designed a lightweight residual network that focuses on the inherent relationships in the spatial domain, aiming to extract key information from images. The structure of LResNet is shown in Fig. 2. In the graph, BN refers to batch normalization and Swish is an activation function. ReLU is a commonly used activation function in neural networks. It is a left-saturated function with a derivative of 1, which prevents the gradient from decaying as quickly as the sigmoid function does, providing the advantage of speeding up training and overcoming gradient extinction. However, when the ReLu input is negative, the output is always 0, resulting in no activation. Swish overcomes the problem that ReLu is invalid when the input is negative^36^. The Swish functional expression is shown in Eq. (3).

3 $$\begin{aligned} f(x)=x\cdot \textrm{sigmoid}(\beta ,x) \end{aligned}$$

Where $\beta$ is a constant.

PQD image generated through convolution operation 112 × 112 × 64 feature diagram. The maximum pooling layer compresses the input feature map and extracts the main features, while enhancing the robustness of the model to some extent. To achieve a lightweight model, this paper uses two deep separable convolutions (GConv) to construct a residual block, which has only 1.73% of the parameter of a standard convolutional residual block. Multiply the feature map from the residual module by the feature map from the previous layer to obtain a new feature map to prevent model degradation. Then, a convolutional layer is used to extract local features of the image. Finally, use GAP to integrate the spatial information on each channel, obtaining a 1 × 1 × 128 spatial feature vectors.

Compared to the traditional ResNet18, LResNet has a simpler structure and 99.25% fewer parameters. Its performance meets the requirements of extracting spatial features for this paper.

#### TFE module

The PQD signal is fed into the TFE module to extract temporal features, and the first requires normalization of the raw data. In this paper, min-max normalization is used to map the perturbed signal x to between [0,1]. The formula is Eq. (4).

4 $$\begin{aligned} x^{*}=\frac{x-min(x)}{max(x)-min(x)} \end{aligned}$$

Then input the normalized data into LSTM for training and extract temporal features. LSTM is a neural network that is improved to solve the problem of gradient vanishing in recurrent neural networks^37,38^. The input gate of LSTM is used to read data, while the forgetting gate discards useless information and preserves valid information. The output gate transmits effective information to the next moment.

The input gate of LSTM is used to read the data and the forgetting gate discards the useless information and keeps the valid information. The output gate delivers valid information to the next moment. The calculation formula are shown in Eqs. (5)–(10)

5 $$\begin{aligned} f_t=\sigma (W_f [h_{t-1},x_t]+b_f) \end{aligned}$$

6 $$\begin{aligned} i_t=\sigma (W_i [h_{t-1},x_t]+b_i) \end{aligned}$$

7 $$\begin{aligned} g_t=\textrm{tanh} (W_g [h_{t-1},x_t]+b_g) \end{aligned}$$

8 $$\begin{aligned} o_t=\sigma (W_o [h_{t-1},x_t]+b_o) \end{aligned}$$

9 $$\begin{aligned} C_t=f_t C_{t-1}+i_t g_t \end{aligned}$$

10 $$\begin{aligned} h_t=o_t \textrm{tanh} (C_t) \end{aligned}$$

At time *t*, $x_t$ is the input of time t, $\sigma$ is sigmoid function, $f_t$ is forgetting gate, $i_t$ is input gate, $g_t$ is output of tanh function, $o_t$ is output gate, $C_{t-1}$ is the carrier of the previous round of global information, $h_{t-1}$ is the intermediate state output of the previous round. $C_t$ is the carrier of this round of global information, $h_t$ is the intermediate state output of this round. $W_f$, $W_i$, $W_g$, $W_o$ are the weights of the corresponding symbols, $b_f$, $b_i$, $b_g$, $b_o$ are the bias of the corresponding symbols.

Due to the different operating speeds of LSTM and LResNet, the time required for the TFE and SFE modules to complete feature extraction is not synchronized. Therefore, the module that completes the extraction first needs to wait for the other module to complete its operation before being input into the MFF layer for feature fusion. Experiments have shown that the TFE module runs faster, and the extracted temporal features need to be stored in memory before the SFE module completes the spatial feature extraction. Usually, data is stored in main memory, waiting for the CPU to issue a call instruction, and then the data is input into the CPU to execute the subsequent program. However, the CPU access to main memory is slow. To speed up the call, we use a cache between the CPU and main memory to store the timing characteristics. When the SFE completes its operation, the CPU reads the timing characteristics data directly from the Cache at high speed, speeding up the program’s execution.

### MFF and classification

**Figure 3:**
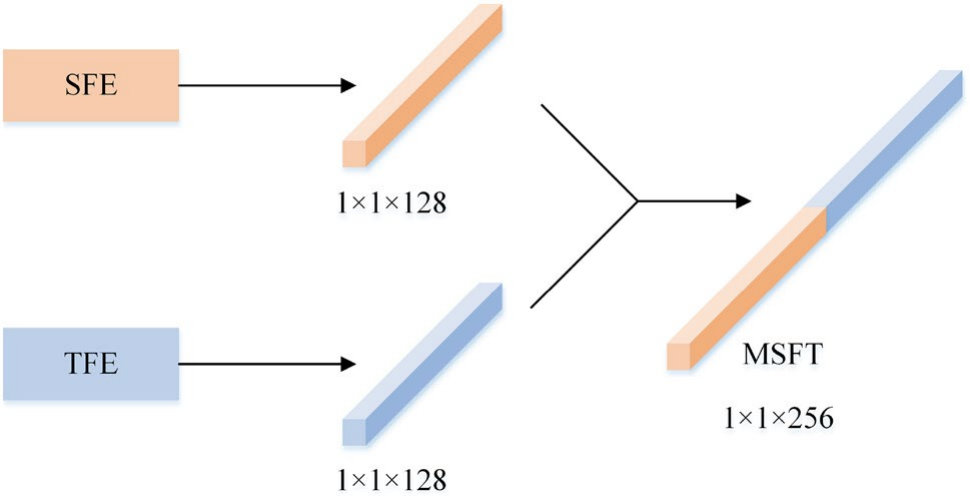
Multimodal feature fusion.

The PQD signal uses SFE to extract a size of 1 × 1 × 128 spatial features, extracted using TFE with a size of 1 × 1 × 128 temporal features. Directly concatenate and fuse two sets of feature vectors to obtain 1 × 1 × 256 is shown in Fig. 3. This multimodal feature vector preserves the spatiotemporal feature information of the PQD signal to the maximum extent through direct concatenation.

SVM is a machine learning algorithm that can handle high-dimensional data, overcome dimensionality catastrophe, has better robustness and interpretability, and has better generalization ability to provide more reliable results. Therefore, SVM is chosen as the classifier in this paper.SVM maps multimodal perturbation features into a high dimensional space by means of a kernel function and searches for an optimal hyperplane in that space to classify the PQD^39^. The classification problem can be transformed into a quadratic programming problem. The objective function and constraints is shown in Eq. (11).

11 $$\begin{aligned} \left\{ \begin{array}{l} \min \frac{1}{2}\Vert \omega \Vert ^{2}+C \sum _{i=1}^{l} \xi _{i} \\ \text{ s.t. } y_{i}\left( \omega x_{i}+b\right) \ge 1-\xi _{i}, i=1,2, \cdots , l \end{array}\right. \end{aligned}$$

In Eq. (11), C is the penalty factor, $\xi _i$ is the relaxation term, $x_i$ is the training data, $y_i$ is the classification label, $\omega$ is the weight matrix, and b is the polarization parameter. The linear kernel function $K(x_i,x_j)=|\varphi (x_i),\varphi (x_j)|$ is used to find the optimal hyperplane through global search, and the optimal classification decision function is obtained as shown in Eq. (12).

12 $$\begin{aligned} f(x)=sgn\sum _{i=1,j=1}^{n}a_{i}^{*} y_j K(xi,x_j)+b^{*} \end{aligned}$$

In Eq. (12), $$a_{i}^{*} $$ and $$b^{*} $$ are solutions of the above formula, n is the number of training samples, $$x_j$$ is the input vector, $$y_j$$ is the corresponding expectation.

## Simulation experiments

### PQD dataset

The mathematical model of the PQD signal is based on IEEE 1159-2019 standard^40^. The mathematical model of a single disturbance is shown in Table 1. A single disturbance superimposes each other to form a composite disturbance. Using MATLAB to call the rand function to generate 20 types of power quality signals with random amplitude and random disturbance occurrence time within the range of parameters and at a sampling frequency of 3 kHz, and add 30 dB white noise to simulate noise interference during the acquisition process. The PQD signal includes normal voltage (S1), sag (S2), swell (S3), interruption (S4), harmonics (S5), transient oscillation (S6), flicker (S7), and transient pulse (S8), sag + oscillation (S9), swell + oscillation (S10), flicker + oscillation (S11), sag + harmonic (S12), swell + harmonic (S13), flicker + harmonic (S14), sag + pulse (S15), swell + pulse (S16), oscillation + harmonic (S17), sag + harmonic + oscillation (S18), swell + harmonic + oscillation (S19), flicker + harmonic + oscillation (S20). There are 300 samples for each type of power quality signal, for a total of 6000 sets of sample data. The sample data is divided into a training set and a testing set at 7:3, and 10% of the training set is taken out for validation.

**Table 1:** Mathematical model of power quality signal.

Signal	Mathematical model	Parameter
S1	$y(t)=A[1\pm \alpha (u(t-t_1)-u(t-t_2))]sin(\omega t)$	$\alpha \le 0.1,T \le t_2-t_1 \le 9T$
S2	$y(t)=A[1- \alpha (u(t-t_1)-u(t-t_2))]sin(\omega t)$	$0.1\le \alpha \le 0.9, T\le t_2-t_1\le 9T$
S3	$y(t)=A[1+ \alpha (u(t-t_1)-u(t-t_2))]sin(\omega t)$	$0.1\le \alpha \le 0.8, T\le t_2-t_1\le 9T$
S4	$y(t)=A[1- \alpha (u(t-t_1)-u(t-t_2))]sin(\omega t)$	$0.9\le \alpha , T\le t_2-t_1\le 9T$
S5	$y(t)=A[\alpha _1 sin(\omega t)+\alpha _3 sin(3\omega t)+\alpha _5 sin(5\omega t)+\alpha _7 sin(7\omega t)]$	$0.05\le \alpha _3,\alpha _5, \alpha _7 \le 0.15\sum \alpha _i^{2}=1$
S6	$y(t)=A[sin(\omega t)+\alpha ^{\frac{-c(t-t_1)}{\tau } }sin\omega _n(t-t_1)(u(t_2)-u(t_1))]$	8ms$\le \tau \le$40 ms,300 Hz$\le f_n \le$900 Hz
S7	$y(t)=A[1+\alpha _f sin(\beta \omega t)]sin(\omega t)$	$0.1\le \alpha _f \le$0.2, 5 Hz$\le \beta \le$20 Hz
S8	$y(t)=A[1-\alpha (u(t-t_1)-u(t-t_2))]sin(\omega t)$	$0\le \alpha _i \le 0.414, T/20 \le t_2-t_1 \le T/10$

### Evaluation indicators

In this paper, accuracy recall rate, precision rate, F1 score and parameters are used as evaluation metrics. The calculation formula is shown in Eqs. (13)–(15).

13 $$\begin{aligned} Recall=\frac{TP}{TP+FN} \end{aligned}$$

14 $$\begin{aligned} Precision=\frac{TP}{TP+FP} \end{aligned}$$

15 $$\begin{aligned} F1{\text{-}}score=\frac{2\times Precision \times Recall}{Precision+Recall} \end{aligned}$$

Where TP denotes true positive. TN means true negative. FP means false positive. FN means false negative.

### Simulation analysis

**Figure 4:**
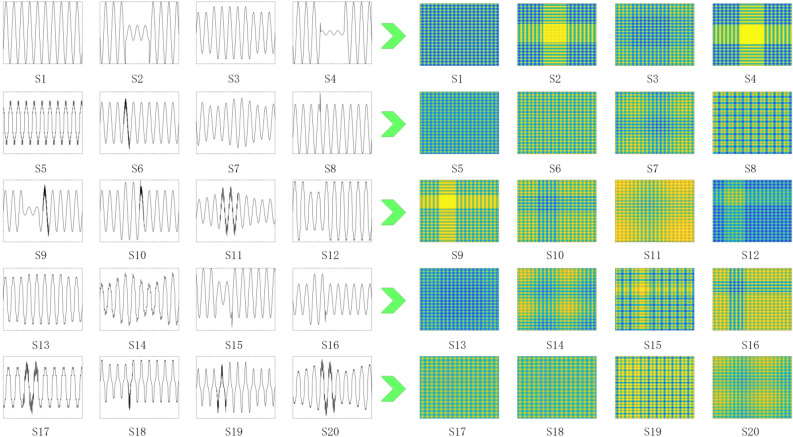
SFE module disturbance image conversion.

The two sub-modules of the multi-modal model only need simple training, and then the output of the prediction intermediate layer is fused through the MFF layer, and finally input into the SVM to realize PQD classification. The SFE module converts the PQD signal into an image using GAF, and the conversion result is shown in Fig. 4.

Set LResNet training for 1 round with a batch size of 30 and a learning rate of 0.001. The number of neurons of LSTM is set to 128, the maximum number of rounds is 30, the batch size is 30, the initial learning rate is 0.001, and the learning rate decreases by a factor of 0.1 every 10 rounds. Both modules use the ‘adam’ optimizer. Using cross entropy as the loss function, the mathematical formula for the function is shown in Eq. (16).

16 $$\begin{aligned} C=\frac{1}{n} \sum _{x}^{} [yln(a)+(1-y)ln(1-a)] \end{aligned}$$

In Eq. (16), y is the expected output and a is the actual output.

**Figure 5:**
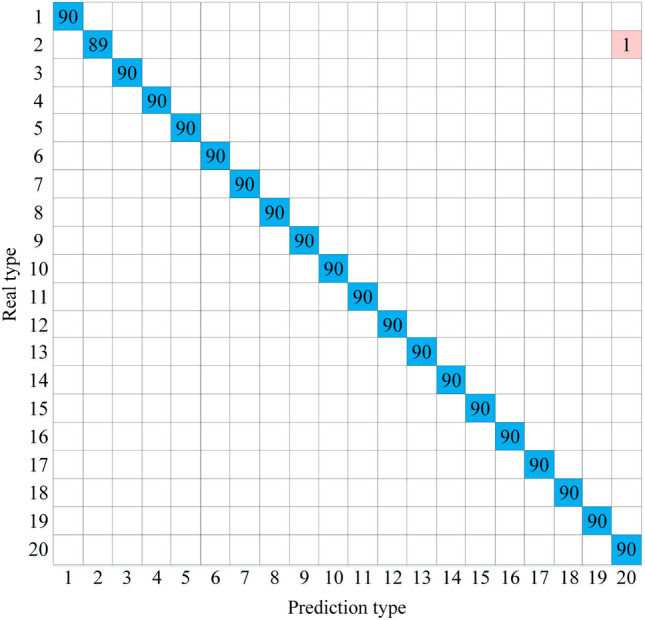
Classification results of multi-modal LResNet-LSTM-SVM.

The confusion matrix is introduced to show the classification results of the model, as shown in Fig. 5.

In Fig. 5, only one S2 (red box) was incorrectly identified as S20 in the test set, and the rest of the types were accurate. To verify the role of each module, ablation experiments were done in this paper, and the experimental protocol and classification results are shown in Table 2.

**Table 2:** Ablation experimental results.

Scheme	Model	Recall (%)	Precision (%)	F1 (%)	Parameters (MB)
1	LSTM	76.94	79.17	78.04	0.07
2	LResNet (ReLu)	82.91	80.22	81.54	0.01
3	LResNet (Swish)	86.44	87.46	86.95	0.01
4	LResNet (ReLu)-LSTM-SVM	99.83	99.83	99.83	0.08
5	LResNet (Swish)-LSTM-SVM	99.94	99.95	99.94	0.08

As can be seen from Table 2, it can be seen that the correct recall rate of Scheme 3 has increased by 6.22% compared to Scheme 2, and the recall rate of Scheme 5 has increased by 0.11% compared to Scheme 4. This verifies that the model using the Swish activation function is better than the model using the ReLu activation function. The correct rates when using LSTM and LResNet alone are only 76.94% and 86.44% due to insufficient feature information extracted by a single modality. The fusion of the features of the two modalities resulted in a significant increase in the correctness rate, which reached 99.94%. Therefore, multi-modal features contain more information than single modal features and can fully grasp the characteristics of PQD signals. And the model does not add too many parameters, only 0.08 MB.

In order to reflect the advantages of multi-modal models, nine single modal models are built for comparison in this paper, namely GRU, AlexNet, GoogLeNet, Xception, ResNet18, ResNet50, ResNet101, EfficientNet-B0, MobileNetV2 and ShuffleNetV1. The same data set was used for the experiments. Calculate the evaluation indicators of the model according to formulas (13–15), and the comparison results are shown in Table 3.

**Table 3:** Comparison of multi-modal and single modal model.

Model	Recall (%)	Precision (%)	F1 (%)	Parameters (MB)
GRU	77.00	77.44	77.22	0.05
AlexNet	83.72	84.59	84.15	43.31
GoogLeNet	76.56	76.48	76.52	5.72
Xception	95.61	96.46	96.03	19.22
ResNet18	97.39	97.61	97.50	10.67
ResNet50	95.22	95.59	95.40	22.53
ResNet101	96.22	96.85	96.53	40.62
EfficientNet-B0	98.11	98.18	98.14	3.87
MobileNetV2	98.33	98.41	98.37	2.30
ShuffleNetV1	98.45	98.44	98.45	1.40
LResNet-LSTM-SVM	99.94	99.95	99.94	0.08

In Table 3, the accuracy of LResNet-LSTM-SVM improved by 22.94%, 16.22%, 23.38%, 4.33%, 2.55%, 4.72%, 3.72%, 1.83%, 1.61% and 1.50%, respectively, over the comparison model. Compared with GRU, the model’s parameter size only increased by 0. 03 MB. The number of parameters in this model is only 0.18%, 1.40%, 0.42%, 0.75%, 0.36%, 0.20%, 2.07%, 3.48% and 5.71% of the other 9 deep learning models. Therefore, the proposed multi-modal model in this paper not only has higher classification accuracy than the single-modal model, but also has less parameters and achieves the lightweight of the model.

### Real data validation

**Figure 6:**
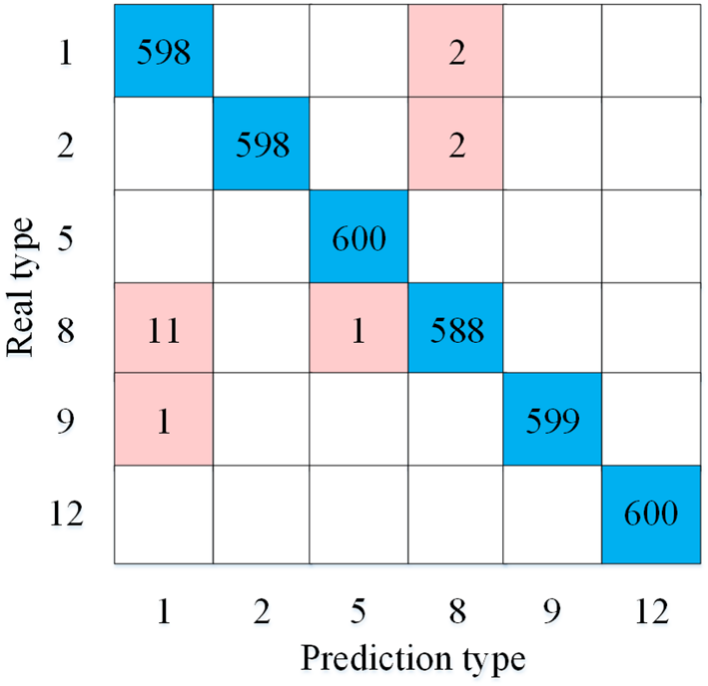
Confusion matrix for real data classification results.

In order to further validate the feasibility of the method, this paper validates the method using a set of real signals as inputs. The dataset is provided by the Kaggle public database and includes six categories of power quality signals, namely normal voltage (S1), sag (S2), harmonics (S5), transient pulse (S8), sag + oscillations (S9), and sag + harmonics (S12). There are 600 samples for each signal type. The confusion matrix of the classification results is shown in Fig. 6.

As can be seen in Fig. 6, 2 groups of S1 are incorrectly identified as S8 due to the influence of noise, 2 groups of S2 are identified as S8 due to smaller amplitude of voltage drop and noise interference, 11 groups of S8 are identified as S1 due to smaller amplitude of pulse, 1 group of S8 is incorrectly identified as S5 due to larger number of pulses in the sample, and 1 group of S9 is identified as S1 due to smaller amplitude of voltage drop and oscillation. Although there is a certain difference between real data and simulated data, the model of this paper still achieves 99.53% classification accuracy, which is only 0.41% less than the simulation results, thus verifying the effectiveness of the method.

## Conclusion

For the problem of PQD classification, this paper proposes a PQD classification model based on multimodal LResNet-LSTM parallel feature extraction.

- The model proposed in this article consists of three modules: PFE, MFF, and classification. The two sub-modules SFE and TFE of PFE are utilized to extract spatial and temporal features in parallel. Then merge the two types of features into a MSTF. Finally, the MSTF is input into SVM for classification. The model can recognize 20 types of PQDs with an accuracy rate of 99.94% and a parameter size of only 0. 08MB.
- A simple-structured Light ResNet was designed based on residuals. Unlike traditional ResNet18, the residual block of LResNet uses two deep separable convolutions, greatly reducing the number of parameters in the model. And LResNet uses the Swish activation function instead of the original ReLu, which optimizes the classification performance of the model.
- This article uses a high-speed cache to address data storage problems caused by asynchronous execution of SFE and TFE, but the capacity of high-speed cache is limited and unsuitable for large-scale data storage. Therefore, we are considering adopting a more appropriate method to control the operation of both modules in the future, enabling them to complete feature extraction simultaneously, and avoiding data transfers and reads.
- Unlike traditional deep learning models that improve classification accuracy by increasing depth, the proposed model in this paper not only improves classification accuracy, but also reduces the number of parameters in the model.

## Data Availability

The simulation dataset generated during and analysed during the current study are available from the corresponding author on reasonable request. Real dataset: https://www.kaggle.com/datasets/aswarthnarayanacv/power-quality-distribution-dataset-2.
